# Mechanistic trials, therapy and developmental science—An exemplar from early autism care

**DOI:** 10.1002/jcv2.70051

**Published:** 2025-10-17

**Authors:** Jonathan Green

**Affiliations:** ^1^ Division of Psychology and Mental Health School of Health Sciences The University of Manchester Manchester UK; ^2^ Manchester University NHS Foundation Trust and Royal Manchester Children's Hospital Manchester UK

**Keywords:** autism, autistic social development, causal inference, developmental outcomes, mechanistic analysis, randomised controlled trials

## Abstract

**Background:**

Mechanistic design and analysis in clinical trials remains relatively rare in child mental health and autism, despite the considerable value that it could have in developing therapy practice and in illuminating basic science. Clinical trials themselves continue to have insufficient influence on actual clinical practice in child mental health.

**Methods:**

Aspects of clinical trial design, including co‐design, are identified that could increase their face validity for clinicians. A number of recent developments in mechanistic analysis of complex intervention trials in mental health are described that can also increase the face validity and utility for clinicians, and provide causal inferences for clinical and developmental theory that are useful transdiagnostically.

**Results:**

These possibilities are exemplified with findings from the Paediatric Autism Communication Therapy (PACT) trial programme in early autism care. This is one of the largest in the field and unique in achieving a long‐term trial follow‐up with preserved intention to treat analysis. Mechanistic analysis in this programme identifies a key cascade of mediation, with therapeutic changes in early child dyadic social initiation mediating generalised endpoint as well as follow up autism outcomes. These findings identified causal inferences regarding the responsiveness of sociability in autistic children to environmental adaptation in therapy, with implications for mechanism across developmental science. They also facilitated successful adaptation of this therapy into other non‐specialist environments.

**Conclusions:**

Well‐designed mechanistic trials identify active processes underlying therapy effects. They have in consequence powerful potential to facilitate therapy adaptation and transdiagnostic refinement. They can also uniquely illuminate developmental theory, by allowing causal inferences in developmental science that are difficult in other ways.

## Key points

**What's known**

- Mechanistic design and analysis in clinical trials can uniquely illuminate theory and clinical practice, but remain relatively rare in child mental health, including in autism.

**What's new**

- The potential of mechanistic analysis is exemplified in a series of early intervention trials in autism, including long‐term follow‐up data.
- These show how autistic sociability can be responsive to environment change, and how autistic children can generalise this to impact long‐term developmental outcomes.

**What's relevant**

- These findings are relevant to understanding early autism development, to early development intervention, and to how therapies can empirically evolve and adapt to new contexts.
- Mechanistic randomised controlled trials pursued in this way can increase their relevance to clinicians and provide illuminating causal interferences for developmental scientists.

## INTRODUCTION

### The mechanistic approach

Mechanistic thinking is implicit in all models of psychological therapy and practice, in the sense that each inevitably contains a theory of mental functioning linked to an idea of the therapeutic processes by which that functioning can be influenced or changed. We can example psychoanalytic therapy, evolving alongside a theory of ‘hydraulic’ and structural interplay of unconscious and conscious mental processes; or cognitive behavioural therapy, alongside a theory of cognitive processes and their link to affect, beliefs and behaviours. Even if not explicit, therapy models likewise will contain an implicit ‘logic model’ reflecting their ‘theory of change’; for instance, the reworking of mental material through transference interpretations in psychoanalysis, the provision of a therapeutic secure base within John Bowlby's attachment therapy, or mechanisms of cognitive re‐structuring in cognitive behavioural therapy. Whilst implicitly central to all therapeutic models, it has however been less common historically for such theoretical mechanisms to be actually tested empirically. Often, the processes involved have been considered just too complex and nuanced for empirical investigation, or such empirical inquiry has been resisted for theoretical reasons and felt to be irrelevant for actual clinical practice. Instead, the evolution of therapies has tended to be marked by personal therapist allegiance to (‘branded’) models and competition for dominance between them; arguably an inefficient and poorly reasoned process for evolving effective therapy. This despite long‐standing conceptual interest dating at least from Jerome Frank (Frank & Frank, 1991), and discussed further below, on what might be the effective elements shared in common across apparently disparate forms of psychological therapy and healing.

More recently, there has been increasing confidence that empirical methods can indeed be applied to the subtleties of psychological treatment and psychotherapy without reductionist oversimplification; and as this confidence has grown, methods have evolved empirically to address therapeutic process and potential common factors. Marchette and Weisz (2017) describe the advantages and advances promised by such empirical approach. They outline how a mechanistic approach points to possibilities beyond self‐contained ‘branded’ therapies with their strong adherents; to a more dynamic intervention science responsive to advances in basic science understanding and working towards refined effectiveness. It is an approach also relevant to stratification and personalisation of therapy. They point to how treatments might work trans‐diagnostically, targeting shared pathological mechanisms across problem areas or diagnostic groups; that treatment implementation could itself become more modular, combining flexibly a number of common active mechanisms across different treatment approaches; and thirdly, at the most general and ambitious level, advocate a ‘*principle‐based approach*’ in which both trans‐diagnostic targets and common elements might be combined into adaptable generic forms of therapy practice. That paper articulates well an argument that will have face validity to many practitioners; that in clinical practice, an informed flexible approach, mobilising a menu of well‐defined strategies in a timely fashion in a particular context, is an intelligent way of delivering evidence‐based practice, personalised to individual young people and families.

### Aims of this paper

Despite the ambitions articulated by Marchette and Weisz (2017), this kind of approach remains the exception not the rule in child development and mental health (Schmidt & Schimmelmann, 2015). Moreover, there can be continuing scepticism within the practitioner community as to the relevance of randomised controlled trial evidence (on which this work fundamentally rests). My aim in this paper, through a worked example in autism, is to advocate for the mechanistic randomised controlled trial (RCT); showing how insights can be gained beyond therapeutic efficacy as to just these theories of mental functioning and change that have characterised the evolution of psychological treatments.

I aim to:

i) Discuss a framing, including co‐design, of clinical trial methods, to improve their relation to clinical experience;
ii) Describe evolving forms of mechanistic analysis within clinical trials and what they promise in terms of illumination of therapy process and background theory;
iii) Present in detail an example of a mechanistic randomised controlled trial methodology within early autism intervention, and how findings from this illuminate both therapy process and also developmental science; in the specific area of early autism development, but also applicable trans‐diagnostically.

## METHODS

### Increasing the relevance and acceptability of the clinical trial

Clinical trials and their methods can still sometimes seem remote to many in the clinical and lay communities, in what can be seen as their abstraction from the complexity of everyday and clinical life. I want firstly to emphasise a beauty in the rigour of trial methods, particularly in their mechanistic design, but also to discuss ways of improving their relevance to everyday life and clinical experience. Just because much in trial methodology involves minimising risks of various forms of bias, including random allocation of subjects as well as standardisation of procedures, there can sometimes be a feeling from clinicians and stakeholders that the method implies a lack of valuing the personal and individual. I argue on the contrary that this method just brackets temporarily such personal considerations at the service of a trial test; which is then in itself at the service of powerfully informing back to individual clinical and personal experience. The quality therefore of how a trial is ‘*located*’ in such contexts becomes of key relevance. This matters at every level of interface with the community; from how questions are initially selected and posed, designs are made and agreed, to participant selection, measurement, interpretation and finally usage of the trial information back into the clinical world. Co‐design from the outset between trialists, clinicians, service‐users and stakeholders across all these elements (Donetto et al., 2015) is now becoming increasingly important for the acceptability and relevance of the clinical trial to the practical world. To this end, the ideal trial can be framed as bringing the reality of complex individual lives, through responsive but rigorous measurement, into a more abstract domain of group commonalities; translated then into a numerical language for analysis. Then after the trial experiment has completed, to translate from those numbers back again, through interpretation within measurement, into outcome inferences about the individual lives that began the process in the first place. The quality of each of these steps determines the quality and acceptability of the outcome inferences.

Initial participant selection for instance is a key decision; the underpowering of many trials reduces their ability to apply back probabilistically from what happens in the trial to the wider population of relevance to stakeholders. Measurement is another key area, foundational for scientific method, yet commonly given insufficient attention or explanation in trials. Measurement has real work to do to capture the essence of a complex psychological reality across individuals in a way amenable to statistical analysis. Poor measurement practice (or what is considered by critics the simple impossibility of measuring the things they are interested in) can feed a sense that clinical trials are such a pale reflection of reality as to be inadequate or unethical as an investigative tool. Equally now though the thoughtful ‘ownership’ of measurement decisions within co‐design models can be crucial to acceptability in the clinical and stakeholder community.

### Mechanistic analysis

The trial process becomes even more vivid with the analysis of mechanism; beyond *whether* an intervention works, to *how* it works. Increasingly sophisticated mechanistic methods for complex intervention trials in mental health (Dunn et al., 2015) allow powerful causal inferences both within the trial but also beyond it in terms of processes in the real world (Green & Dunn, 2008). They bring alive the maxim that ‘the best way of understanding a system is to try to change it’.

#### The ‘hybrid trial’, moderated mediation and inferences for theory

One extension of the mechanistic approach was described by Howe et al. (2002). They outline how a combination of random parallel group allocation differentially exposed to a theoretically targeted intervention, coupled with an observational repeated measures follow‐up design, forms what they call the ‘hybrid prevention trial’, which can be used as a powerful method for investigating mechanism over time. Well‐constructed interventions create a targeted ‘developmental perturbation’ leading to downstream effects that allow true causal inferences to be drawn; something not possible from simple longitudinal observational studies, however sophisticated the statistical modelling. Dunn et al. (2015) developed further these analytic techniques; addressing issues of measurement error and post‐randomisation confounds to improve estimations, the moderation of mediation effects, and the use of instrumental variables. These techniques allow a more accurate modelling of real‐world clinical complexity, giving trials an increased face validity for clinicians and greater relevance for informing clinical theory (Green & Dunn, 2008; Marchette & Weisz, 2017).

#### New techniques of mediator estimation

**FIGURE 1:**
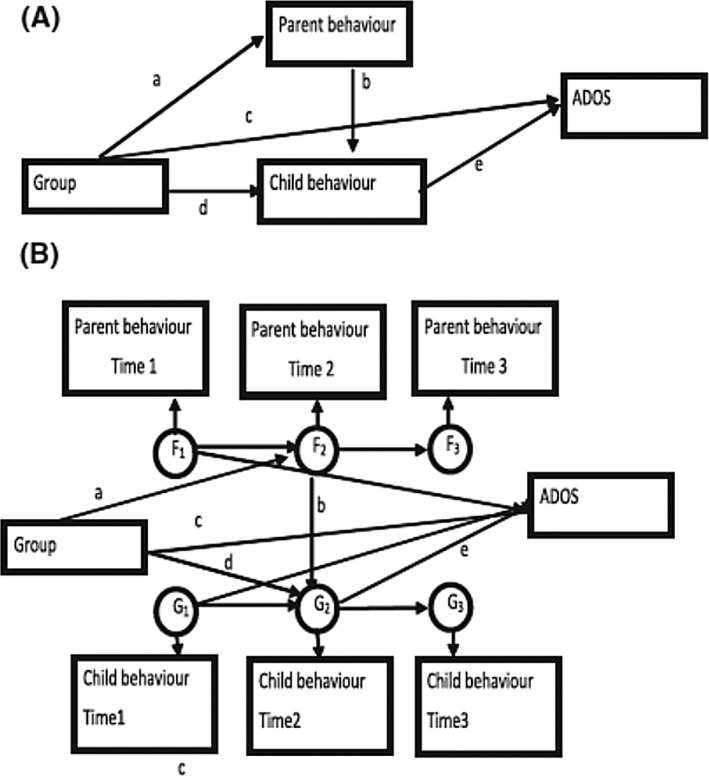
New techniques of latent mediator estimation using repeated measures, used in the PACT mediation analysis (see text). Full mediation models in the PACT analysis are for treatment effect via parent and then child dyadic behaviour on the outcome child autism‐related behaviours (measured on ADOS); (A) Naive model, (B) repeated‐measures model. (Reproduced from (Pickles et al., 2015)). ADOS, Autism Diagnostic Observation Scale; PACT, Paediatric Autism Communication Therapy.

New techniques for mediation analysis have also developed, utilising the repeated measures estimations described above in the hybrid trial. Thus, in a technique used the Paediatric Autism Communication Therapy (PACT) analysis detailed below in this report (Carruthers et al., 2023; Pickles et al., 2015), repeated measurement of the putative mediator over time, alongside its inter‐rater reliability, is used to estimate a latent mediator variable, which when applied in analysis reduces the effect of measurement error on the mediator and increases the precision of mediation estimation, as shown in Figure 1.

#### Caveats

As the use of mediation analysis has grown, there have been technical arguments in favour of undertaking mediation analysis even if there is no primary outcome effect (MacKinnon et al., 2007). However, despite the important learning there may be from such analyses, a caveat arises if publication of positive mediator effects on non‐significant endpoint outcomes is made in the absence of a separate formal report of the null effects of the trial itself (e.g., Yoder et al., 2021). Since from this, a misleading impression could be given that the therapy is actually effective; whereas clearly a mediator cannot be used as a surrogate outcome in such a context. Protection against such potentially misleading impressions can simply come from good trial reporting practice; pre‐registration of the trial and analyses, a sequenced publication plan, outcome reports that contain complete primary and secondary data outcomes and are transparent on null results; and reporting all these separately from the mechanistic analysis itself.

#### Descriptive alternatives

Such approaches need to be distinguished from a *descriptive* form of ‘common elements’ approach that is not truly mechanistic. For instance, Chorpita and colleagues (Chorpita & Daleiden, 2009; Chorpita et al., 2005) undertook a systematic descriptive classification of therapy elements found to be common across a number of efficacious therapies in a particular area. Their implication was that these ‘common elements’ may represent key active ingredients; and that an integrated approach could be refined by emphasising them. Kasari & Smith (2013) also advocated for the value of identifying ‘key components’ of a therapy in education that were essential; alongside other elements where there could be more flexibility for adaptation to different environments. Chorpita's approach is attractive and indeed can distil down shared elements across different therapeutic approaches; however there are obvious flaws in the logic. Firstly, there is the third factor confound in the fact that similarities in the content of these different therapists could simply derive from common theoretical bases rather than common effectiveness. They do not address the mechanistic question of whether these common elements are *actually* those that have an effect on outcome. The approach can then end up just being circular. More concerning, and increasingly frequent, is when, assuming that these identified ‘common elements’ ensure effectiveness, new therapies utilising some of these elements thereby claim effectiveness without any of their own testing. The descriptive approach has clear value in distilling down common candidate elements for mechanistic effect, but an essential next step has then to be test these candidate elements in more experimental paradigms.

## RESULTS

### An exemplar of the PACT mechanistic trial and its inferences

The PACT trial tested the Paediatric Autism Communication Therapy, a parent‐mediated intervention specifically designed to address the early developmental precursors of social communication, social engagement and language relevant to the emergence of autism in the early years of childhood.

#### Background context

Traditionally, intervention for early autism was focused on applications of behavioural learning theory, which had the aim of ‘normalising’ what were seen as atypical or abnormal behaviours associated with autism, using behavioural modification techniques focused on extinguishing specific unwanted behaviours and promoting normative alternatives. These interventions are applied directly to the child by a therapist or therapy team (Lovaas & Koegel, 1973). The PACT form of therapy grows out of a different ‘transactional’ (Sameroff, 2009) and developmental approach to autism; an idea of how heritable autistic variation within neurodiversity emerges as the autism phenotype in the first few years of life, through developmental unfolding but also within early transactional relationships in the environment (Green, 2022). Empirical developmental science work in infancy underpins this idea. In our initial longitudinal study of infants at increased familial likelihood (HL) of an autism trajectory (*N* = 45) compared to low likelihood community controls (LL, *N* = 45), parent‐infant interaction was studied in a standardised play setting through video analysis on the Manchester Assessment of Caregiver‐Infant Interactions (MACI) (Wan et al., 2013). Differences in interactions between HL and LL groups could be seen from as early as 7 months infant age, with increased caregiver ‘directiveness’—actions that shape and lead the child's responses in a way that controls the interchange and reduces sensitive responsiveness and reciprocity (usually motivated by an anxiety on the caregiver's part that the child will not be able to manage and needs to be shaped and held). At 14 months these caregiver differences continued, with the addition now of emerging differences in *infant* interactions, with reduced attentiveness to caregiver, affect and mutuality. The differences in infant interaction at 14 months were then shown to be correlated with a later autism diagnosis at 3 years: infants who went on to develop autism specifically showed, at 14 months, significantly reduced attentiveness (adjusted *F* 6.95, *p* < 0.01), positive affect (*F* 7.31, *p* < 0.01), and mutuality (*F* 6.5, *p* < 0.01) (Wan et al., 2013). These findings, confirmed in subsequent studies (Wan et al., 2019) represent one of the earliest specific markers of later autism development. We interpret them as consistent with a transactional model of early functioning; in a cascade effect where the presence of neurodiversity or pre‐autism in the infant shows measurable effects on parental response within the dyad at 7 months, generalising to both infant and parent by 14 months, indicating a trajectory that anticipates later autism diagnosis at 3 years. Here then is a form of theory of change model mentioned in the introduction. Consistent with this is an intervention support model that aims to mitigate these early dyadic phenomena, and thus enhance later infant developmental trajectory outcomes (Green, 2022). The logic model of PACT therapy acts in just this way.

#### PACT logic model

**FIGURE 2:**
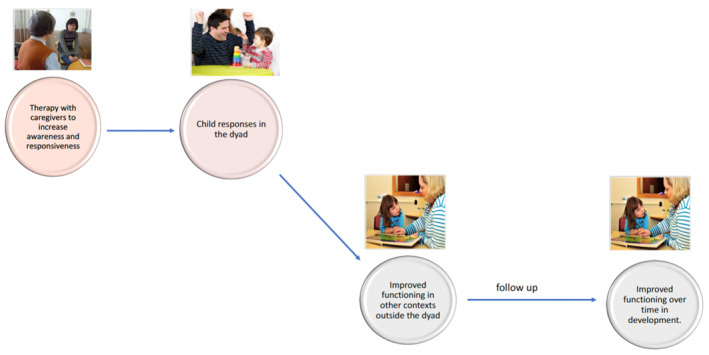
The logic model of the Paediatric Autism Communication Therapy.

Instead of working directly with the child with the goal of altering specific aspects of behaviour, PACT therapy works exclusively with and through the parent or caregiver, with the aim of altering the ongoing naturalistic inter‐personal and transactional environment around the child in early development. This work with parents, centrally using video‐feedback technique, is designed to help their awareness and understanding of the particularities of the communication style and intent from their neurodivergent child, to improve the accuracy, sensitivity and contingency of their dyadic responses (Aldred et al., 2018). The logic model of the therapy (Figure 2) is then that this improvement in adult attunement and responsiveness will initiate an alteration in the interpersonal transactional dynamics between child and adult; leading in theory to an increase in the child's spontaineous social engagement and communication; and re‐balancing the dyadic interaction towards reciprocity. This inter‐personal ‘re‐balancing’ would reflect what has been called a ‘coupling’ in early social interaction (De Jaegher & Di Paolo, 2007), of a kind central to naturalistic social learning in neurotypical development (Tomasello, 2008). PACT therapy is manualised and developmentally staged to build on this early dyadic synchrony towards further social and communication engagement. The second stage in the logic model is that this alteration in dyadic interaction between parent and child will lead to more ‘distal’ generalised improvement in child behaviour, social orientation and motivation across context and through time as they grow. Such a second stage could involve ‘within‐child’ processes familiar from neurotypical development science, but less well studied in autistic development. For instance, adapted forms of procedural learning and symbolic representation to allow domains of related skill (e.g., in social communication) to be applied flexibly across contexts, or as a developmental cascade from precursor skills through to developmentally related subsequent cross‐domain abilities or adaptations (Crank et al., 2021). Such a second stage is likely also however to involve more general psychological processes linked to the initial dyadic work: such as enhanced child emotional and relational security and co‐regulation, leading to enhanced epistemic trust, confidence, and improvement in self‐regulation of behaviour and arousal and reduced internal distress. The presence of this latter mechanism is supported by qualitative parent‐report data from our trials (Leadbitter et al., 2020). It provides a plausible explanation for the cross‐domain impact we see downstream on reduced anxiety and the intensity of restricted and repetitive behaviours (RRB; Carruthers et al., 2023); since these latter are often associated with distress arousal. (It is worth noting that autistic RRB's can be non‐disruptive and associated too with pleasure and excitement, in ways we would not expect or wish to be influenced by this therapy). Much of this has not been enough directly studied or demonstrated in autism; but, as will be seen below, we do get suggestive indications on it from our mechanistic studies.

#### Measurement

The extent to which the parent is able successfully to understand and respond to the child in the way envisaged in the therapy, is measured through an assessor‐blinded video coding of a parent‐child free play episode taken separate to the therapy context. Reflecting the empirical research in infancy (Wan et al., 2019), there are codes of the proportion of parental ‘synchronous responses’ to the child, and, independently, the proportion of child behaviours that are ‘social communication initiations’ to the parent. The generalised within‐child developmental change later in time was measured in our trials firstly with an assessor using the standard Autism Diagnostic Observation Scale (ADOS); an overall summary scale of the full range and richness of autistic‐related developmental behaviours, objectively codable and with a modular structure allowing comparable measurement through development. The ADOS has been subject to recent concern and criticism (e.g., Timimi et al., 2019), for legacy issues, from its early development three decades ago, of deficit‐focused language and assumptions in its coding. Moreover, because it is commonly used as one part of clinical diagnostic assessment, an altered ADOS dimensional score can be sometimes linked to concerns about (categorical) autistic essence or identity. These are valid issues and improvements need to be considered. However, as used in this paper, I propose that the phenomena coded in ADOS can be understood and valued as inherently developmental (see Green, 2022). In good hands, the instrument is sensitive to the autistic child and brings out both strengths and vulnerabilities within autistic difference. It has extensive external validity for development in longitudinal studies into adulthood, and, in our trials and beyond, the ADOS score is responsive to developmental change and co‐varies with other developmental phenomena such as parent‐ and teacher‐rated adaptative competencies, parent rated communication, language, reduction in interfering sensory behaviours (Carruthers et al., 2023; Pickles et al., 2016), and parent‐nominated priority outcomes (Leadbitter et al., 2017). The measure is not perfect, but has a large corpus of data associated with it which I hope to show, can illuminate strengths‐based developmental gains, such as for instance more effective communication and social reciprocity, reduced disruptive stimming and behavioural rigidity (these latter improvements we hypothesise being the result of improved communication and arousal regulation), as well as increased adaptive flexibility.

#### Intervention effect

**FIGURE 3:**
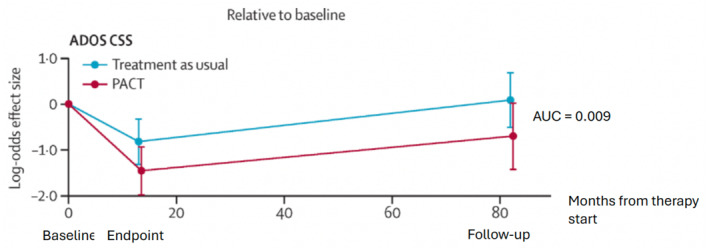
Primary outcome from the PACT parent‐mediated intervention trial at endpoint and 6‐year follow‐up. Shows intervention effect to improve a combination of social communication skills, repetitive behaviour, and sensory sensitivities (ADOS calibrated severity score measure), with improvements sustained from endpoint to 6 years after the end of therapy. AUC = area between curves estimation over time (see text); this estimate provides a principled basis for the overall mean effect on unequally spaced measures that summarises treatment effect over the whole trial period from baseline to follow up. (Reproduced from Pickles et al., 2016). ADOS, Autism Diagnostic Observation Scale; PACT, Paediatric Autism Communication Therapy.

A first small RCT of the PACT intervention compared to usual care was designed in this fashion (Aldred et al., 2004) and sampled 28 pre‐school children aged 2–5.11 years with a diagnosis of ‘core’ autism. It found a substantial treatment effect on outcome child ADOS total score (*F* 1,25 = 7.30; *p* = 0.01), a result particularly carried by the therapy effect to increase competency in social communication. In the subsequent larger MRC PACT RCT (*N* = 152; Pickles et al., 2016), also with pre‐school core autism, we found a significant intervention endpoint effect on the full autistic phenotype (social communication and ‘restricted repetitive behaviour’ including sensory domains considered together); OR −6.4; −1.22, −0.06, *p* = 0.02. This endpoint treatment effect was then sustained over time: in follow up intention‐to‐treat (ITT) analysis 6 years after initial treatment end, with 80% of the original cohort assessed at a mean age 10.5 years, the between‐group treatment effect on ADOS scores was preserved (OR −8.2; −1.53, −0.12, *p* = 0.02), giving a highly significant cumulative effect of therapy (marginal log‐odds effect size of 0.55 [95% CI 0.14–0.91]; *p* = 0.009; shown as an ‘area between curves’ AUC, Figure 3). Parallel sustained change was also seen in parent‐reported outcomes in relation to communication, adaptation and family functioning; and teacher‐ratings of adaptive function in school. One area not showing change was objectively‐measured ‘structural’ language (vocabulary and grammar), although parent reported vocabulary and communication increased.

#### Mechanism of intervention effect

**FIGURE 4:**
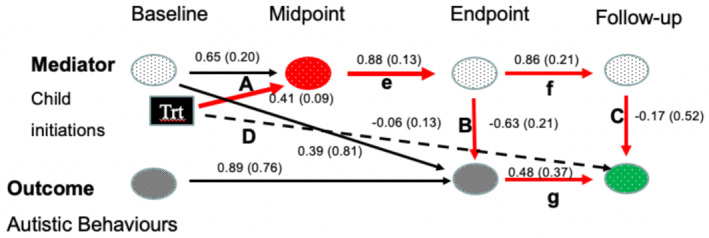
Standardised structural path coefficients (standard errors) between mediator (child initiations) and the primary outcome of autism behaviours measured on the ADOS calibrated severity score measured at endpoint and 6‐year follow‐up. Squares are observed variables, ovals are latent variables, single‐headed arrows are direct effects. Dashed line D is the non‐mediated direct effect of treatment on the outcome at follow‐up, while paths A, B and C fall on paths of indirect effects between treatment and outcome at follow‐up. (Adapted from Carruthers et al., 2023). ADOS, Autism Diagnostic Observation Scale.

Mechanistic analysis of both these trials identified the mediating (or ‘active’) processes at different stages of the therapy towards achieving these outcomes. In the first trial the increased parental synchrony from treatment mediated the ADOS change at the end of therapy (Aldred et al., 2012). In the second larger trial a more sophisticated latent mediator modelling was used (Figure 1), identifying a three‐stage mediation process (Figure 4):

1) within the immediate parent‐child dyad (the ‘proximal’ target of therapy), improved parental ‘synchrony’ strongly mediated an increase in spontaineous child communication initiations with parent (Pickles et al., 2015);
2) this increase in child dyadic communication initiations in turn strongly mediated the later improvement in ADOS generalised outcome at therapy endpoint (Pickles et al., 2015);
3) these same improvements, in child dyadic communication initiation during the intervention period, also strongly mediated the sustained reduction in autism‐related behaviours found at 6 years follow up in middle childhood; with 73% mediation through midpoint child initiations (path: A × e × (B × g + f × C) = −0.16, CI −0.42, −0.05), and 27% through a direct effect via path D (Carruthers et al., 2023).

Essentially the same mediation pathway also accounted for a near‐significant increase in teacher‐rated functional outcome in school for the intervention children at the 10.5 years follow up (Carruthers et al., 2023). The total effect on follow‐up here was small but positive (ES = 0.14 on the mediation variable, bootstrap CI −0.02, 0.27, Wald *p* = 0.052). This mediation effect was divided almost equally (55% mediated) between direct (ES = 0.06, CI 0.25–0.22) and indirect effects (ES = 0.08, CI 0.07–0.41).

**FIGURE 5:**
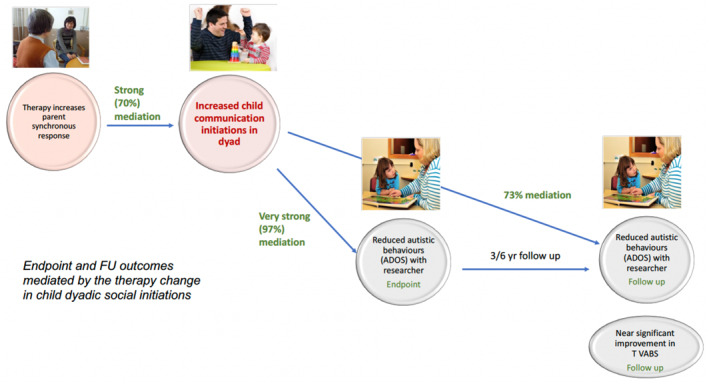
Summary of mechanistic findings in Paediatric Autism Communication Therapy.

We thus here identify successive stages in the timing of effects: the first immediate short‐term effect on *dyadic interaction* of increased parental understanding, responsivity and ‘synchronous’ communication are to increase the child's spontaneous social initiation and engagement. This evidences the intended emergent ‘coupling’ of social interaction (De Jaegher & Di Paolo, 2007), which is also marked by an increase in manifest shared enjoyment and parent reports of ‘light‐bulb’ moments of connectedness (often for the first time) with their child (Leadbitter et al., 2020). Such a finding is consistent with much of what we know about how dyadic interaction works in neurotypical social development. What is new here (and consistent too with findings from some other relationship‐focused interventions in this field [Gulsrud et al., 2016; Mahoney & Solomon, 2016]) is to find that neurodivergent children also respond in a similar way, with increased social engagement. Subsequently, we identify a secondary generalisation of this short‐term dyadic change into longer term impact on child social communication, behavioural and adaptive outcomes in development, both at therapy endpoint and then sustained at a 6‐year follow‐up. The path analysis (Figure 4) identifies this as first a generalisation to functioning outside the family at therapy endpoint; including improvements in both social communication and RRB domains. Then it identifies a combination of sustained dyadic child initiation effects, in parallel with a continuation of the endpoint developmental social communication and behavioural effects, over the next 6 years. These are remarkable findings in long‐term downstream causal impact from dyadic change in the original therapy. We did not here have measurement to investigate the within‐child processes through which the dyadic change generalises to phenotypic change outside the family. We did however show in a later study (Green et al., 2022), that such mediation was not impacted by child variables of ‘resistance to change’ or developmental quotient. While objective change in ‘structural language’ is not seen, there is sustained improvement in the ‘pragmatics’ of discourse, with this latter acting as a key interactional accomplishment for the child that can increase their connectedness with others (Sterponi et al., 2015). Parent reports of wider developmental improvement in language and family functioning (Leadbitter et al., 2017) also substantiate the broader improvements. These mediation results overall support the logic model of the PACT parent‐mediated intervention, as how it is intended to work. But they have further developmental implications for a transactional model of autism development, as I will develop further below. The central conclusion from these mechanism analyses, therefore, is that increase in child spontainous social engagement (initiations) during therapy substantially causes the significant downstream developmental changes witnessed at endpoint and follow up (Figure 5).

A third related mechanistic analysis involved an adaptation of the original clinic‐based PACT therapy to be suitable as a multicomponent intervention simultaneously in the home with parents and in education/school with learning support assistants (PACT‐G; Green et al., 2022). In this trial, *N* = 249, the primary autism behaviour outcome (ADOS and related BOSCC measure) failed to show a significant treatment effect. The mechanism analysis however showed preservation of a significant ‘proximal’ Step 1 effect in parental synchrony and child communication in dyadic interaction, across both home and school (albeit at a reduced effect size to the original PACT trial). Some evidence for the Step 2 generalisation from child initiation to behavioural outcome was still present but at a much weaker level and just on the BOSCC (Fig. S2 in Green et al., 2022). The reason for only a partial mechanistic replication we put down to the reduced dosage in each context in the PACT‐G model, the complexities of implementation in education and also at home, evidence by reduced model fidelity, and a possible effect of the increased proportion of online delivery in this therapy iteration. From this we infer important learnings around dosage thresholds and attending to the implementation context.

## DISCUSSION

### Implications of the mechanistic findings

#### Intervention practice

The long‐term mechanistic findings within the PACT RCT are to our knowledge unique in the autism intervention literature and uncommon in developmental science practice more generally (but see Brody et al. (2016), for an example in a different field). In practice terms, the mediation findings on PACT support its logic model: the targeted change in parental function achieved by the therapy has the theorised consequence of improving child dyadic social initiation and engagement. Further, the child initiation change generalises into change in child autistic developmental and functional behaviours outwith the therapy and family context and over time. This is shown significantly in autistic behavioural outcomes, as well as with near‐significant effects on school functioning, 6‐years after the end of therapy. It is a unique strength then of the RCT method that these mediation results allow truly causal inferences; in a way really impossible to do otherwise in developmental or observational research. The mechanistic inferences within the therapy show in detail *how* the therapy works, and this turns out here to be in a way that is consistent with the related developmental science (findings convergent with those seen also in Mahoney & Solomon, 2016).

Knowing this active mechanism then gives purchase on the kind strategies outlined by Marchette and Weisz (2017), at the head of this article:

1. To refine and make more efficient the intervention delivery, by concentrating on aspects that promote the active effects and downplaying others. For instance, the mechanism findings across PACT and PACT‐G indicate that achieving a strong effect‐size in proximal parental synchrony change is critical, and is maximally enabled by the core video‐feedback technique, probably ideally in a clinic‐based setting. Additionally, the fact that the maximal effect on the mediating variable of child initiations occurred after 6 months of the initial 12 month therapy, gave logic for a shortening of therapy length in some contexts. On the other hand, the reduced mechanistic transmission in the combined home/school context of PACT‐G suggests that there are likely to be critical session dosage thresholds for achieving effectiveness, and that the environmental conditions in which a therapy is given can make a substantive difference to overall outcomes.
2. To allow adaptations to the model for other contexts, while preserving the active ingredients and processes. For instance, using a systematic co‐design model in South Asia (Divan et al., 2015), we developed a culturally adapted version of PACT (PASSPlus). This preserved the core elements of PACT (including the video‐feedback procedure linked to our mechanistic outcomes in UK trials), whilst introducing acceptable variations that made non‐specialist delivery feasible and appropriate for these different cultures in which there was a shortage of specialist practitioners. Subsequent RCTs have shown that non‐specialist delivery of this adapted PASSPlus does replicate the key dyadic effects found in UK PACT (Divan et al., 2019; Rahman et al., 2016) and, in a large scale‐up trial (*N* = 261, Roy et al., 2023), despite a null primary endpoint, precisely the same mechanistic pathway as originally identified above in PACT (Divan et al., 2025). To show such common dyadic and developmental processes across very different cultural contexts is an important and suggestive outcome for psychological therapy; illuminating for both theory and practice. Nevertheless, such common effects across culture also have to be understood alongside the major environmental differences in health care and culture that remain (Divan et al., 2021), and which are also key to understand in adaptation work.
3. To represent a step towards more integrated and efficient modular interventions, for autism and even trans‐diagnostically, which utilise demonstrated active mechanisms of this kind, along with the therapeutic elements linked to them, in improved adaptive combination.

#### Developmental science

Strong inferences for theory also arise from this mechanistic data. Autistic children are shown to respond, in a causal sense, to an adapted, more attuned and responsive, social environment, through an increase in their own spontaneous social initiations. Although this may not seem so surprising in the context of neurotypical development, it actually contradicts much previous autism theory and has significant implications:

1. It gives empirical evidence against a fully essentialist notion of ‘social impairment’, currently definitional within the autistic phenotype. It suggests the reality is more contextual; consistent with a position increasingly advocated in the theoretical literature (Jaswal & Akhtar, 2019), in phenomenological study with autistic people (Murray et al., 2022), from other relationship‐based intervention trials (Mahoney & Solomon, 2016), and theory of the transactional emergence of the autistic phenotype (Green, 2022). In straightforward terms, ‘sociability’ in the autistic child can be thought by this as not simply an intrinsic impairment but something more contextually and transactionally determined. The mechanistic findings provide causal evidence for this, since by changing the interpersonal environment we see a consequent change in sociability. By better understanding autistic neurodivergence within this transactional frame, we have a logic with which to adapt and adjust other social environments in support of autistic development and flourishing (Green, 2022).
2. The *spontaineous* nature of the child social initiations measured here gives this behaviour a quality of ‘agency’ (i.e., coming *from* the child rather than imposed *on* them). This is a particularly timely given the discussions within neurodiversity advocacy and theory on the ethics of any intervention support in autistic adults and children; that these may be an imposition or encourage a ‘masking’ of autistic reality in a neurotypical world (a particular concern with behavioural learning methods). The kind of adapted environment of caregiver attention and attunement aimed for in interventions like PACT works differently and is empirically supported as a procedure more in tune with autistic community preference (Bent et al., 2024).
3. The second step of the mediation process in PACT demonstrates how the increase in social initiation within the dyad is generalised into change in domain‐related autistic behaviours across the range of the autistic phenotype, extending over time. This suggests a true developmental generalisation of effect. It is in keeping with the way that developmental science indicates is central in neurotypical childhood; that early dyadic interactions are internalised into mental or cognitive schema which themselves link to change in domain‐related behaviours in other contexts. It is important to note that this is not generalisation of specific behaviours across contexts, a phenomenon often discussed in the behavioural literature (Carruthers et al., 2020). It is rather the procedural learning or generalisation of a class of behaviours (Hong et al., 2019) related to both social functioning and restricted, repetitive and sensory behaviours. In this way the autistic child also appears to be able to generalise acquired skills and schema within procedural learning, something that theory had often suggested the autistic child could not do.

## CONCLUSION

Mechanistic intervention science works towards establishing shared generalisable knowledge of therapeutic effects, which can be a counterpoint to simple adherence to specific theories or therapies and be incorporated into a more flexible evidence‐based personalised practice. There is a reciprocal interplay here with basic developmental science and the results from observational methods (Bottema‐Beutel, 2025). Thus, if an intervention is grounded in related developmental theory and science, and if clinical trials can identify active mechanisms underlying its therapeutic efficacy; then there can be an illumination in return of key causal aspects in that underlying developmental theory, in a way that I have argued only a mechanistic RCT with repeated measures follow‐up can do. Causal demonstration of the way autistic sociability (and other aspects of the phenotype) respond to environmental change provides evidence to modify previous developmental theories. Moreover, the downstream generalisation of acquired skill across domain suggests the possibility that autistic children too likely show procedural learning and developmental schemas in a way that had often been thought not possible, opening up new areas for investigation. A further benefit has been to use this mechanistic understanding in adapting the principles of therapy into radically different cultural contexts and within non‐specialist delivery, showing preservation of efficacy and mechanism in those contexts.

As Marchette and Weisz (2017) argued, this kind of mechanistic understanding can then stimulate further evolution in intervention, and adaptation into across contexts. For instance, the findings on the causal influence of interpersonal environment on child social function, and the generalisation of these effects across context and over time, are relevant to general developmental theory as applicable transdiagnostically to other areas of psychopathology in addition to the developmental theory of autism. My argument has been that only by mounting rigorous trial methodology over time, and in a way that is understandable and acceptable to the clinical and wider community, can we achieve such a result. In this way the mechanistic RCT within complex intervention can prove its value beyond a specific context.

## AUTHOR CONTRIBUTIONS

**Jonathan Green**: Conceptualization; methodology; data curation; investigation; formal analysis; funding acquisition; writing—original draft; writing—review and editing; project administration; validation; supervision.

## CONFLICT OF INTEREST STATEMENT

J. G. declares director fees for a not‐for‐profit community interest company IMPACT (CiC: 10902031) to deliver/disseminate training on the PACT intervention method.

## ETHICAL CONSIDERATIONS

Ethical approval for the PACT trial was granted from the Central Manchester Multicentre Research Ethics Committee (Ref 05/Q1407/311; 9/3/2006); and for the PACT 7–11 Follow‐up study from the North West—Greater Manchester North Research Ethics Committee (Ref 13/NW/0144; 4/3/2013). Ethical approval for the PACT‐G trial was granted from the North West—Greater Manchester Central Research Ethics Committee (Ref 15/NW/0912; 28/1/2016). Written consent to participate was provided by at least one parent in each family enrolled in each study. Images in Figures 2 and 5 are stock images retrieved from public domain sources.

## Data Availability

The data that support the findings of this article are available from the author on request.
